# Rapamycin suppresses postnatal muscle hypertrophy induced by myostatin-inhibition accompanied by transcriptional suppression of the Akt/mTOR pathway

**DOI:** 10.1016/j.bbrep.2018.12.009

**Published:** 2019-01-21

**Authors:** Dong hyuck Choi, Jinzeng Yang, Yong Soo Kim

**Affiliations:** a Department of Molecular Bioscience and Bioengineering, University of Hawaii, Honolulu, HI 96822, USA; b Department of Human Nutrition, Food and Animal Sciences, University of Hawaii, Honolulu, HI 96822, USA

**Keywords:** Myostatin, Propeptide, MTOR, Rapamycin, Transgenic mice, Mrf4

## Abstract

Myostatin (MSTN) is a well-known negative growth factor of muscle mass, and studies have shown that MSTN-inhibition would be a potential strategy to treat muscle atrophy seen in various clinical conditions. Recent studies suggest that MSTN-inhibition induces skeletal muscle hypertrophy through up-regulation of the anabolic Akt/mTOR pathway. Therefore, it was hypothesized that the muscle hypertrophy induced by MSTN-inhibition would be suppressed by the administration of rapamycin (RAP), a mTOR suppressor. A MSTN transgenic mouse strain (MSTN-pro), which is characterized by a postnatal hyper-muscularity due to MSTN inhibition through transgenic overexpression of MSTN propeptide, was used in producing experimental animals. Five-week-old male heterozygous MSTN-pro mice and wild-type littermates were administered with 0 or 3 mg/kg body weight of RAP intraperitoneally every other day for 4 weeks. The effects of RAP on muscle growth, mRNA abundance of signaling components of the Akt/mTOR pathway, and myogenic regulatory factors (MyoD, Myf5, MyoG, and Mrf4) were examined in comparison to wild-type mice. Body weight gain of MSTN-pro mice was significantly greater than that of wild-type mice. RAP suppressed body weight gain and muscle mass in both MSTN-pro and wild-type mice. The extent of both body weight and muscle mass suppression was significantly greater in MSTN-pro mice than in wild-type mice. Real-time qPCR analysis showed that mRNA abundance of the signaling molecules of the Akt/mTOR pathway, including Akt, p70S6K, and 4E-BP1, were significantly higher in MSTN-pro mice. RAP treatment decreased mRNA abundance of Akt, p70S6K and 4E-BP1 only in MSTN-pro mice. mRNA abundances of MyoD and MyoG were not affected by MSTN suppression or RAP treatment. mRNA abundance of Myf5 was decreased by RAP, but not affected by MSTN suppression. mRNA abundance of Mrf4 was decreased by MSTN suppression. RAP treatment decreased mRNA abundance of Mrf4 only in wild type mice. Results of this study indicate that transcriptional regulation of signaling components of the Akt/mTOR pathway and myogenic regulatory transcription factor Mrf4 is involved in the enhancement of skeletal muscle mass induced by MSTN suppression.

## Highlights

• Muscle mass increase induced by myostatin inhibition was suppressed by rapamycin administration.
• Myostatin suppression enhanced mRNA abundance of Akt, p70S6K and 4E-BP1 in mice.
• Rapamycin decreased the expression of Akt, p70S6K and 4E-BP1 in mice with myostatin suppression.

## 1 Introduction

Myostatin (MSTN), also known as a growth and differentiation factor-8 (GDF-8), is a member of the transforming growth factor-beta (TGF-ß) superfamily and negatively regulates skeletal muscle mass [1], [2]. Studies have shown that dysfunctional mutations of the MSTN gene have brought a dramatic increase in muscle mass in cattle [3], [4], [5], dogs [6], sheep [7] and humans [8]. MSTN regulates muscle growth by controlling muscle fiber numbers during the embryonic and fetal development, as well as by controlling the size of muscle fibers postnatally [9]. Like other members of the TGF- ß superfamily, MSTN signals through Smad complex activated by heteromeric complexes of type I and type II serine/threonine kinase receptors. MSTN binds predominantly to activin type IIB receptor (ActRIIB) and two type I receptors, ALK4 or ALK5 [2], [10], leading to the phosphorylation of two intracellular mediators, Smad2 and Smad3 [11], [12]. The phosphorylated Smad2/3 oligomerizes with Smad4, then regulates the transcription of target genes [13]. Even though the Smad signaling pathway activated by MSTN has been well established, the molecular mechanism(s) by which Smads activation regulates skeletal muscle development and growth is not fully elucidated.

Mechanistic target of rapamycin (mTOR) is a key regulator of cell growth, integrating signals from growth factors, nutrients, and energy status to control protein synthesis and other cell functions [14]. Akt is an upstream regulator of mTOR, and various studies in vitro and in vivo have shown that the Akt/mTOR pathway plays a crucial role in muscle growth [15], [16], [17]. Recent studies indicate that there is a crosstalk between MSTN signaling and the Akt/ mTOR pathway via Smad mediation. In myotube cultures, MSTN abundance or suppression decreased or increased Akt phosphorylation, respectively [18], [19], [20]. In mice, over-expression of MSTN decreased the phosphorylation of Akt as well as other Akt/mTOR signaling components, including TSC2, ribosomal protein S6 and 4E-BP1 [21], while knockout of MSTN function increased the phosphorylation of Akt, S6 and S6 kinase (S6K) [19], [22]. A study indicates that Smad activation is involved in the MSTN regulation of the Akt/mTOR pathway [23]. In that study, muscle fiber atrophy was induced in mice by transfection with constitutively active (c.a.) ALK4/5, the type I MSTN receptors. Suppression of Smad 2 and 3 through the siRNA method completely blocked the c.a. ALK4/5-mediated atrophy. Furthermore, the induction of Akt could completely block the atrophic action of c.a. ALK4/5, indicating a presence of crosstalk between the Smad and Akt signaling.

Myogenic regulatory factors (MRFs), including MyoD, Myf5, MyoG (myogenin), and Mrf4, play a key role in skeletal muscle formation and development. MyoD and Myf5 are involved in muscle determination, while MyoG plays a downstream role of muscle cell differentiation to form muscle fibers, and Mrf4 is regarded to be involved in the maintenance of postnatal muscles [24]. Studies have shown that MSTN controls myoblast proliferation and differentiation via regulation of MRFs during prenatal myogenesis. *In vitro* muscle cell culture, inhibition of myoblast proliferation by MSTN was associated with either decreased MyoD [11], [18] or MyoG expression level [25]. In doubled-muscle cattle, which expresses non-functional mutant MSTN, fetal skeletal muscle expressions of MyoD and MyoG were significantly up-regulated [26]. MRFs also play a role in the growth and maintenance of postnatal skeletal muscle, but little is known about how MSTN influences MRFs in association with the regulation of postnatal skeletal muscle growth.

A previous study showed that transgenic mice with depressed MSTN function by skeletal muscle-specific expression of its propeptide cDNA had dramatic skeletal muscle growth and mass [27]. The transgenic mouse (MSTN-pro) colony has been maintained for 17 years, and the muscle phenotypes are stably inherited for many generations (unpublished data). In the current study, we hypothesized that if the regulation of muscle mass by MSTN is via inhibition of the Akt/mTOR pathway, the muscle hypertrophy of the MSTN-pro mice will be suppressed by the administration of rapamycin (RAP), a mTOR suppressor. Currently, little information is available regarding the effect of RAP on postnatal muscle growth induced by MSTN inhibition. This study, thus, examined the effect of RAP on muscle growth, mRNA abundance of signaling components of the Akt/mTOR pathway, and MRFs in MSTN-pro mice in comparison with wild-type mice.

## 2 Materials and methods

### 2.1 Animals and sample collections

All the procedures of animal care were approved by the Institutional Animal Care and Use Committee at the University of Hawaii (protocol #, 13–1597). Mice were housed in Small Animal Facility, UH Manoa with a 12 h light/dark cycle and maintained in a temperature- and humidity-controlled condition. Mice had free access to feed (Lab Diet # 5001 Rodent Diet, 10% kcal fat, ME 3.85 kcal/g, Purina Mills, Richmond, IN) and clean water. An MSTN transgenic mouse strain (MSTN-pro), which overexpresses MSTN propeptide driven by MLC1 promoter, was used in producing experimental animals [27]. The MSTN-pro strain is characterized by a postnatal hyper-muscularity due to MSTN activity suppression by the overexpression of MSTN propeptide [27]. Wild-type male B6SJL F1 mice and MSTN-pro female mice (B6SJL F1) were mated to produce heterozygote MSTN-pro and wild-type littermate genotypes. Pups were weaned at 4 weeks of age, and tail tissues from male pups were collected at the time of weaning for PCR-genotyping. After genotyping, male mice were separated by their genotypes, and each genotype divided into two groups (0 or 3 mg/kg body weight of rapamycin). Rapamycin (RAP) administration started at 5 weeks of age. 400 μg of RAP was dissolved in 10 μL of DMSO, and the RAP stock in DMSO was suspended with 990 μL of PBS. RAP was intraperitoneally (i.p.) administered every other day for 4 weeks, and body weight was measured twice a week. At the end of the administration, animals were sacrificed by CO_2_ asphyxiation, and gastrocnemius, plantaris, and soleus muscles were collected, weighed, and frozen in liquid nitrogen and stored at –80 °C until analysis. Heart, liver, kidney, spleen, and epidydimal fat were also collected and weighed.

### 2.2 PCR-genotyping

DNA of the tail tissue was extracted by phenol/chloroform extraction after overnight digestion at 50 °C in a Tris buffer (1 M, pH 8.0) containing 10% SDS, 0.5 M EDTA, and proteinase K (0.7 mg/mL). Polymerase chain reaction (PCR) was performed with a primer set specific for transgenic mice, and the presence of a transgenic PCR product was examined following the procedure described previously [28].

### 2.3 Real-time quantitative PCR (qPCR)

Total RNA was isolated from individual plantaris muscle samples using the Trizol reagent (Invitrogen, CA, USA) following the manufacturer's protocol. Briefly, Trizol was added to individual plantaris muscle samples (0.5 mL/mg wt), homogenized and left for 5 min at room temperature. 0.2 mL of chloroform per 1 mL of Trizol was added and vortexed for 15 s. After incubation for 3 min at room temperature, the samples were centrifuged at 12,000×*g* for 15 min at 4 °C. The upper layer of the aqueous phase was transferred into a new tube, and 0.5 mL of 100% isopropanol was added. After the incubation for 10 min at room temperature, the samples were centrifuged at 12,000×*g* for 10 min at 4 °C. The supernatant was removed from the tube, and the RNA pellet was washed with 1 mL of 75% ethanol. After vortexing briefly, the tube was centrifuged at 7500×*g* for 5 min at 4 °C. Ethanol was discarded, and the pellet was air-dried for 5–10 min. The RNA pellet was suspended with 50 μL of RNase–free water. Total RNA was treated with 1 μL of RNase free- DNase I (Thermo Scientific, Hudson, NH, USA) to prevent contamination from any DNA. Total RNA was used to generate the cDNA by using Transcriptor Reverse Transcriptase (Roche Applied Science, Mannheim, Germany). Ten μM of Oligo (dT)15 primer was added to 1 μg of RNA with final volume being adjusted to 20 μL by adding DNase-, RNase-free water, and incubated at 65 °C for 10 min. After incubation, 4 μL of 5 × Transcriptor RT Reaction Buffer, 2 μL of 10 mM dNTP, and 0.5 μL of Transcriptor Reverse Transcriptase were mixed well and incubated for 30 min at 55 °C. The activation of Transcriptor Reverse Transcriptase was stopped by incubating at 85 °C for 5 min. Synthesized cDNA was ten times diluted with nuclease-free water and ready for real-time qPCR.

The expression level of genes, including MyoD, Myf5, MyoG, Mrf4, Akt, 4E-BP1, p70S6k, and MSTN was estimated by the real-time quantitative PCR using the above cDNA. The sequences of primer sets of each gene were designed to yield ~150 bp and are shown in Table 1. The primer sets were validated for their robustness by the presence of a single band in PCR, single peak in dissociation curve in real-time PCR, and efficiency determination. Real-time PCR reaction was prepared with SYBR® Select Master Mix (Applied Biosystems, Foster City, CA). Each reaction included 2 μL of diluted cDNA, 10 μL of 2x SYBR Master Mix, 0.5 μL of 10 p.M. primer, and Nuclease-free water. The real-time qPCR was run on Applied Biosystems 7300 Fast Real-Time PCR (Applied Biosystems, Foster City, CA) following the manufacturer's instruction. The cycling conditions were 1 cycle of denaturation at 94 °C for 4 min, and amplification step was performed as above for 42 cycles: 94 °C for 40 s, 60 °C for 30 s, and 72 °C for 30 s. In all the qPCR, amplification of GAPDH sequence was used for endogenous reference gene. The comparative Ct method was used for data analysis. The level of the target gene was normalized with endogenous GAPDH reference gene by subtracting the Ct values of respective GAPDH from the Ct values of the target gene, which is designated as ΔCt Value, or relative gene expression level [29]. Ct values for GAPDH ranged between 15 and 17, and the Ct value for GAPDH was not affected either by genotype or RAP administration. Ct values for other genes ranged between 22 and 32. ΔΔCt values were calculated by subtracting mean ΔCt value of target gene from wild-type mice, then fold change for each gene was calculated using the 2^-ΔΔCt^ method.

**Table 1:** Sequence of PCR primers used for real-time PCR.

**Gene**	**Ref Seq**	**Primers (5′-3′)**		**Tm**	**Amplicon size (bp)**
Gapdh	NM_001289726	Forward	ACCCAGAAGACTGTGGATGG	59.0	171
		Reverse	CACATTGGGGGTAGGAACAC	58.2	
MyoD	NM_010866	Forward	GACAGGGAGGAGGGGTAGAG	60.1	219
		Reverse	TGCTGTCTCAAAGGAGCAGA	59.0	
MyoG	NM_031189	Forward	CTGCCTAAAGTGGAGATCCTG	57.8	149
		Reverse	TGGGAGTTGCATTCACTGG	57.7	
Mrf4	NM_008657	Forward	CCCTACAGCTACAAACCCAAG	58.3	146
		Reverse	GCTGAGGCATCCACGTTTG	59.5	
Myf5	NM_008656	Forward	AGGAAAAGAAGCCCTGAAGC	58.1	151
		Reverse	GCAAAAAGAACAGGCAGAGG	57.6	
Akt	NM_001331107	Forward	GCCCTCAAGTACTCATTCCAG	58.1	142
		Reverse	ACACAATCTCCGCACCATAG	57.7	
P70S6k1	NM_001114334	Forward	TGAGTCAAGCCTTGGTCGAG	59.7	125
		Reverse	AAGAGTCGAGAGAGACGCCC	61.0	
4E-BP1	NM_007918	Forward	CGGAAGATAAGCGGGCAG	58.0	149
		Reverse	CAGTGTCTGCCTGGTATGAG	57.7	
Myostatin	NM_010834	Forward	TGCAAAATTGGCTCAAACAG	55.6	182
		Reverse	GCAGTCAAGCCCAAAGTCTC	59.1	

### 2.4 Statistical analysis

All values were expressed as mean ± SEM, and *P* values less than 0.05 were defined as statistically significant. The effects of genotype, RAP, and genotype × RAP interaction on body and muscle weights, as well as the levels of gene expression were analyzed by the Two-way analysis of variance (ANOVA) followed by the Tukey's multiple comparison test for comparison of the means of each group, using Prism 6 software (GraphPad, San Diego, CA).

## 3 Results and discussion

### 3.1 Effects of RAP on body, muscle and organ weights of MSTN-pro and wild-type mice

**Fig. 1:**
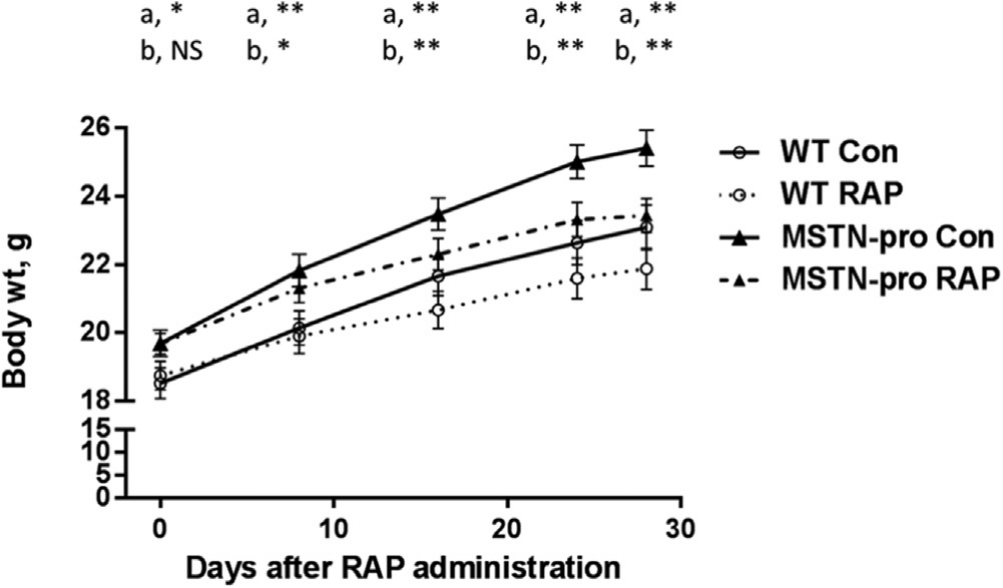
Rapamycin administration suppresses body weight gain of both wild-type (WT) and MSTN-pro mice. Solid and dotted lines indicate 0 and 3 mg/kg RAP administration, respectively, to wild-type (open circle) and MSTN-pro mice (closed triangle). Values are means (±SEM). a, difference between genotype; b, difference between RAP administration (**, P < 0.01; *, P < 0.05; NS, not significant).

The effect of RAP administration on body weight is summarized in Fig. 1. As expected, MSTN-pro transgenic mice grew significantly faster than wild-type mice during the 4 weeks experimental period without RAP administration. Body weight gain of wild-type mice with or without RAP during the 4 weeks period was 17% and 23%, respectively, and body weight gain of MSTN-pro mice with or without RAP was 18% and 33%, respectively. This result confirmed that RAP administration suppressed animal growth in both the wild-type and MSTN-pro mice. In wild-type mice, the growth suppression by RAP was 6% while the suppression was 15% in MSTN-pro mice, showing that the extent of growth suppression by RAP administration was greater in MSTN-pro than that in wild-type mice. Notably, the body weight of MSTN-pro mice at 2 weeks of RAP treatment was not significantly different from that of wild type mice not treated with RAP (Table 2), suggesting that the increased body mass by MSTN is primarily due to the Akt/mTOR pathway, a signaling pathway suppressed by RAP.

**Table 2:** Muscle and organ weights in wild-type and MSTN-pro mice as affected by RAP administration.

	**Wild type mice**		**MSTN-pro mice**		**Significance**		
	**0 mg/kg**	**3 mg/kg**	**0 mg/kg**	**3 mg/kg**	**GT**	**RAP**	**GT × RAP**
# of animals	11	10	7	7			
Initial body wt, g	18.6^a^ (0.36)	18.8^a^ (0.41)	19.4^b^ (0.47)	19.4^b^ (0.30)	*	NS	NS
Final body wt, g	23.5^a^ (0.55)	22.2^a^ (0.47)	26.1^b^ (0.47)	22.8^a^ (0.53)	***	***	NS
Muscle wt^1^, mg	256.0^a^ (7.50)	239.7^a^ (7.25)	410.4^b^ (19.89)	314.3^c^ (24.41)	***	***	**
% Muscle	1.12^a^ (0.017)	1.12^a^ (0.023)	1.46^b^ (0.039)	1.42^b^ (0.072)	***	NS	NS
Heart wt, mg	128.6^a^ (5.34)	126.4^a^ (7.79)	147.2^a^ (5.07)	126.9^a^ (8.88)	NS	NS	NS
% Heart	0.56^a^ (0.018)	0.59^a^ (0.031)	0.53^a^ (0.015)	0.58^a^ (0.030)	NS	NS	NS
Liver wt, g	1.33^a^ (0.031)	1.46^a,b^ (0.049)	1.59^a^ (0.103)	1.34^b^ (0.083)	NS	NS	*
% Liver	5.81^a^ (0.092)	6.81^b^ (0.142)	5.65^a^ (0.274)	6.05^a^ (0.178)	**	***	NS
Spleen wt, mg	71.8^a^ (3.22)	58.6^a,b^ (6.20)	70.6^a^ (4.15)	47.5^b^ (4.80)	NS	*	NS
% Spleen	0.31^a^ (0.013)	0.26^a,b^ (0.024)	0.25^a,b^ (0.013)	0.21^b^ (0.016)	**	*	NS
Kidney wt, mg	454.6^a,b^ (11.52)	420.1^a^ (12.31)	492.3^b^ (16.98)	424.0^a^ (24.05)	NS	*	NS
% Kidney	1.98^a^ (0.056)	2.01^a^ (0.076)	1.84^a^ (0.043)	1.92^a^ (0.070)	*	NS	NS
EP fat^2^ wt, mg	200.9^a^ (26.09)	303.8^a^ (42.18)	212.4^a^ (23.69)	227.1^a^ (55.23)	NS	NS	NS
% EP fat	0.87^a,b^ (0.108)	1.41^a^ (0.159)	0.76^b^ (0.085)	1.03^a,b^ (0.224)	NS	*	NS

Values are means (±SEM). NS, not significant. GT, genotype; RAP, rapamycin. In addition to Two-way ANOVA, means of each group were also compared by the Tukey's multiple comparison test. The means not sharing the same superscript differ at P < 0.05.

* P < 0.05.

*** P < 0.001.

** P < 0.01.

1 Weights of combined soleus, plantaris and gastrocnemius muscles of both legs.

2 Epidydimal fat.

Similar to our result, RAP administration for 2–3 weeks suppressed body weight gain in growing rats [30], [31]. In contrast, others reported that RAP did not affect body weight during a longer treatment (6 weeks to 5 months) in mice [32], [33]. The administration protocol of RAP in these studies (4 mg/kg body weight, i.p. every other day or 2.24 mg/kg body, i.p. daily) was similar to that of our study, but these studies treated mice at 3 or 9 month-old while we treated mice at 5 week-old. Given that 3 month-old mice are already at their mature size, and 5 week-old mice are at the peak of growing, it is likely that RAP suppresses body weight gain of growing animals, while it has little effect on body weight of animals at mature size.

In Table 2, the effects of RAP on muscle and organ weights are summarized. As expected, muscle weight (soleus, plantaris, and gastrocnemius combined), as well as percentage (%) of muscle weight to body weight of MSTN-pro mice were significantly greater than those of the wild-type. RAP significantly suppressed muscle weight in both groups. The suppression of muscle weight by RAP in wild-type mice was 6.4%, while the suppression in MSTN-pro mice was 23.4%, showing that the extent of muscle weight suppression by RAP was greater in MSTN-pro mice than in wild-type mice. Weights of soleus (predominantly type I fiber), plantaris (predominantly type II fibers), and gastrocnemius (mixed type I and type II fibers) were all suppressed by RAP administration (Supplementary Table 1), suggesting that different muscle fiber types were not differentially affected by RAP. In support of the result, a study reported that activation of the RAP-sensitive Akt pathway induced hypertrophy of both soleus and extensor digitorum lognus muscles in adult rats [16].

RAP administration had little effect on the percentages of heart and kidney weight to body weight in both genotypes. A previous study also reported that 1-week administration of RAP did not affect heart weight in 12-week old mice [34]. The percentage of liver to body weight increased significantly by RAP in both genotypes. A study also reported an increase in the percentage of the liver by 2 weeks administration of RAP in 3 month-old mice [32], while other study found that liver size and its ratio to body weight were not affected by 1-week administration of RAP in 12-week-old mice [34]. RAP administration significantly decreased the percentage of the spleen to body weight in both genotypes, in agreement with other results showing decreased spleen size by RAP in various experimental conditions [35], [36]. The percentage of epidydimal fat to body weight increased significantly by RAP in both genotypes. In contrast, many other studies reported that chronic RAP administration reduced adiposity [30], [31], [37]. In 10 week-old rats fed normal or high-fat diet, RAP administration for 3 weeks significantly suppressed fat mass [30], and 16 weeks of RAP treatment also significantly suppressed fat mass and percentage to body mass in 24 week-old mice fed high-fat diet [37]. It currently is not clear what underlying factors or experimental differences could cause the discrepancy in the impact of RAP on fat deposition between our study and others. The discrepancy probably suggests a presence of complex pathways and/or physiological conditions in association with the involvement of RAP on lipid metabolism.

### 3.2 Effects of RAP administration on gene expression levels of myogenic regulatory factors (MRFs)

**Fig. 2:**
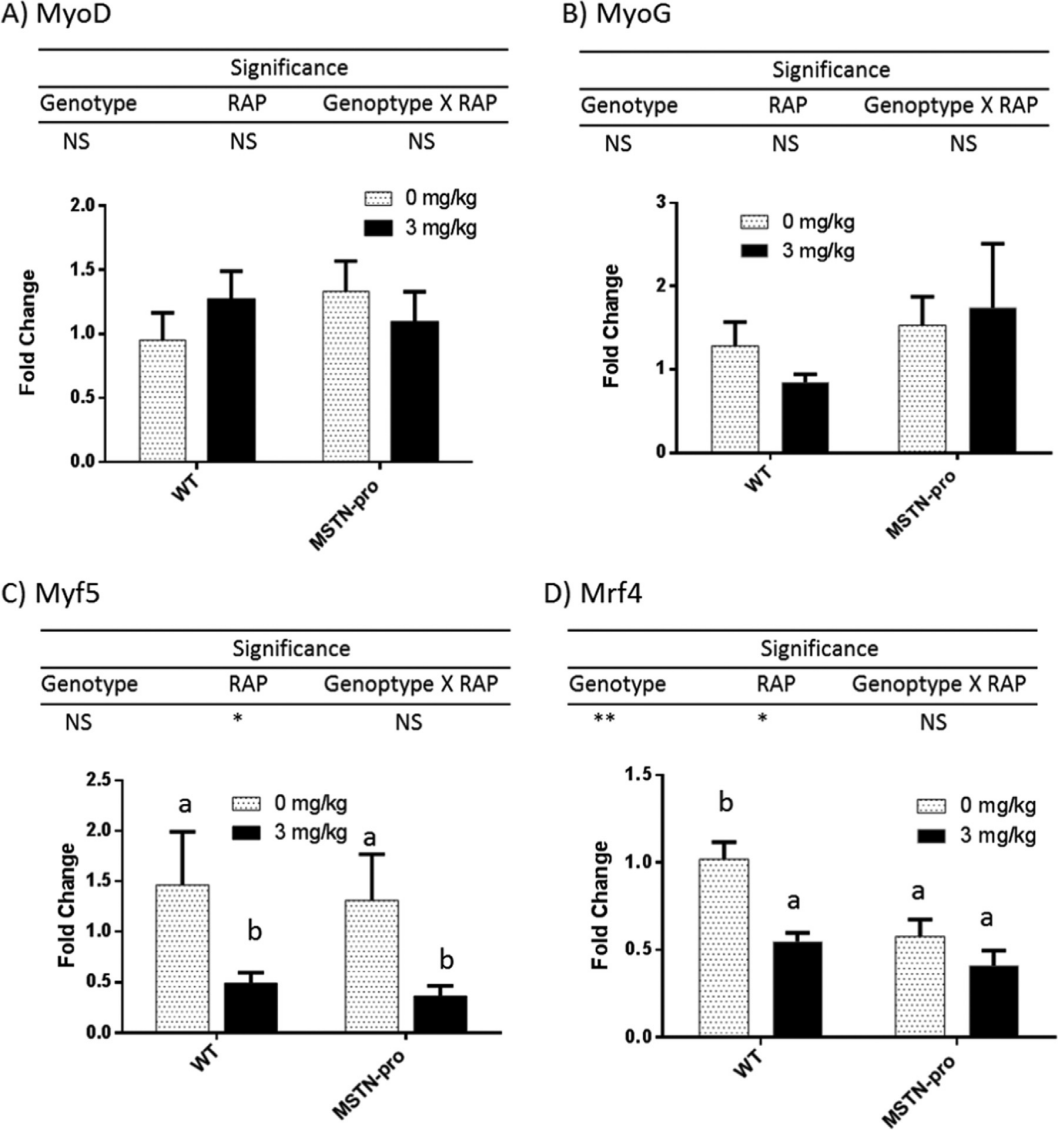
Rapamycin administration suppresses the expression of Myf5 and Mrf4 genes. Gene expression was measured from individual muscle samples. ΔCt values were calculated by subtracting respective CT value of GAPDH from the CT values of the target gene, then ΔΔCt values were calculated by subtracting mean ΔCt value of target gene from wild-type mice, followed by fold change calculation using the 2^-ΔΔCt^ method. The fold changes are expressed as mean ± (SEM). NS, not significant; **, P < 0.01; *, P < 0.05; NS, not significance. RAP, rapamycin. In addition to Two-way ANOVA, means of each group were also compared by the Tukey's multiple comparison test. The means not sharing the same superscript differ at P < 0.05.

The mRNA abundances of four MRFs in MSTN-pro and wild-type mice as affected by RAP are shown in Fig. 2. There was no difference in mRNA abundances of Myf5, MyoD, and MyoG between the MSTN-pro and wild-type mice, whereas mRNA abundance of Mrf4 was significantly lower in MSTN-pro mice than that in wild-type mice. RAP administration did not affect mRNA abundances of either MyoD or MyoG in both MSTN-pro and wild-type mice, while RAP administration significantly reduced mRNA abundance of Myf5 and Mrf4 only in wild type mice but not in MSTN-pro mice.

Transcription factors Myf5, MyoD, and MyoG plays an important role in myoblast determination and differentiation during muscle formation [24]. Therefore, transcriptional regulation of these genes is more prominent during prenatal myogenesis than during postnatal muscle growth. Satellite cells proliferate and differentiate in postnatal muscles in response to the demand of myofiber hypertrophy during growth or enhanced hypertrophy caused by various physiological stimuli, or muscle regeneration [38]. Once satellite cells are activated, they will express MRFs, including Myf5 and MyoD [38]. In this regard, it is possible that increased expression in Myf5 or MyoD is being observed in hypertrophic muscles in postnatal muscles due to satellite cell activation. However, studies have shown that MSTN-induced skeletal muscle hypertrophy occurs independently of satellite cell proliferation and differentiation [23], [39], [40], suggesting that elevated activation of Myf5, MyoD, or MyoG may not be required during the postnatal muscle hypertrophy induced by inhibition of MSTN. The absence of effects on mRNA abundance of MyoD, Myf5, and MyoG by MSTN propeptide suppression on MSTN function in this study is in line with the above findings. Most studies that examined gene expression in animals with the loss of function of the MSTN gene using microarray analysis also reported that MSTN did not affect mRNA abundance of MRFs in mice or cattle [41], [42], [43], while increased expression of MyoG was observed in MSTN-suppressed mice [44].

Interestingly, RAP administration decreased mRNA abundance of Myf5 in both genotypes. We did not measure Pax7 mRNA or protein levels in this study, but it is still tempting to speculate that the decrease in Myf5 transcription was probably associated with a decrease in Pax7/Myf5+ satellite caused by down-regulated muscle hypertrophy upon RAP administration. As far as we know, there has been no study regarding the influence of RAP on the expression of MRFs, and future molecular and cellular studies probably need to examine how the MRFs are regulated by RAP administration during myoblast proliferation and differentiation.

Unlike Myf5, MyoD, or MyoG, Mrf4 is highly expressed after birth and known to play a major role in the maintenance of skeletal muscles [24]. The current result also shows that Mrf4 expression (ΔCt value, ~6.5) is much higher than the expressions of Myf5 (ΔCt value, ~16), MyoD (ΔCt value, ~11) or MyoG (ΔCt value, ~11). In support of a positive role of Mrf4 in the postnatal maintenance of skeletal muscles, studies have shown that Mrf4 expression is down-regulated during disuse atrophy or elevated during hypertrophy induced by stretch or high level of methionine administration in mice or chicken [45], [46], [47]. Contrary to this understanding of Mrf4 as a positive regulator in postnatal muscle maintenance, a recent study showed that Mrf4 suppression via RNAi method induced muscle hypertrophy and prevented denervation-induced atrophy in rats [48], indicating a role of Mrf4 as a negative regulator of postnatal muscle hypertrophy. If Mrf4 indeed is a negative regulator of muscle growth, the decrease in mRNA abundance of Mrf4 in MSTN-pro mice in this study suggests that MSTN potentially up-regulates the Mrf4 to suppress muscle hypertrophy. However, the decrease in mRNA abundance of Mrf4 by RAP administration in wild-type mice does not fit into the newly-proposed negative role of Mrf4 for muscle growth but fit into the positive role for muscle growth. Currently, no clear explanation is available for the conflicting result. Since we did not measure the protein level of Mrf4, we cannot rule out the possibility that the mRNA abundance was not correlated with the level of protein. Nevertheless, conflicting result at the mRNA level appears to indicate a potential presence of complex Mrf4 signaling networks to regulate muscle hypertrophy and maintenance. Thus there is a need for future studies to clearly understand the role of Mrf4 in postnatal muscle growth and maintenance.

**Fig. 3:**
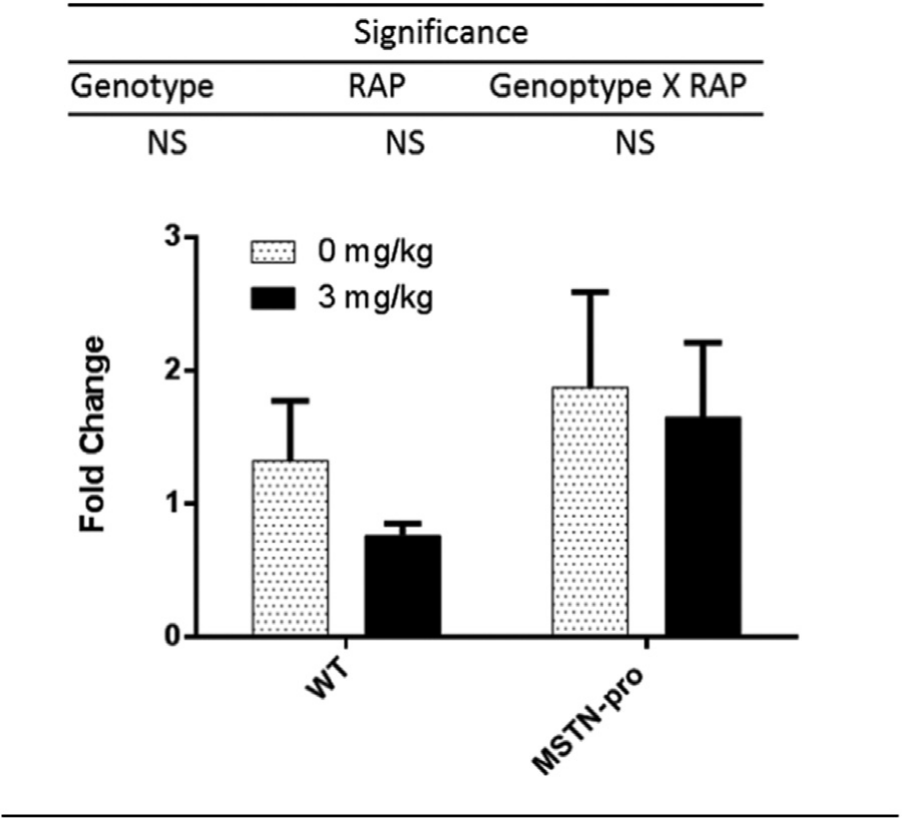
Expression of MSTN in wild and MSTN-pro type mice as affected by RAP administration. Gene expression was measured from individual muscle samples. ΔCt values were calculated by subtracting respective CT value of GAPDH from the CT values of MSTN gene, then ΔΔCt values were calculated by subtracting mean ΔCt value of MSTN gene from wild-type mice. The ΔΔCt values are expressed as mean ± (SEM). Values are expressed as mean ± (SEM). NS, not significant; RAP, rapamycin. In addition to Two-way ANOVA, means of each group were also compared by the Tukey's multiple comparison test.

The mRNA abundance of MSTN in both genotype as affected by RAP is shown in Fig. 3. MSTN gene expression was not affected by either genotype and RAP administration, indicating that RAP is not involved in the regulation of MSTN gene expression.

### 3.3 Expression levels of signal molecules of the Akt/mTOR pathway in MSTN-pro and wild-type mice as affected by RAP

**Fig. 4:**
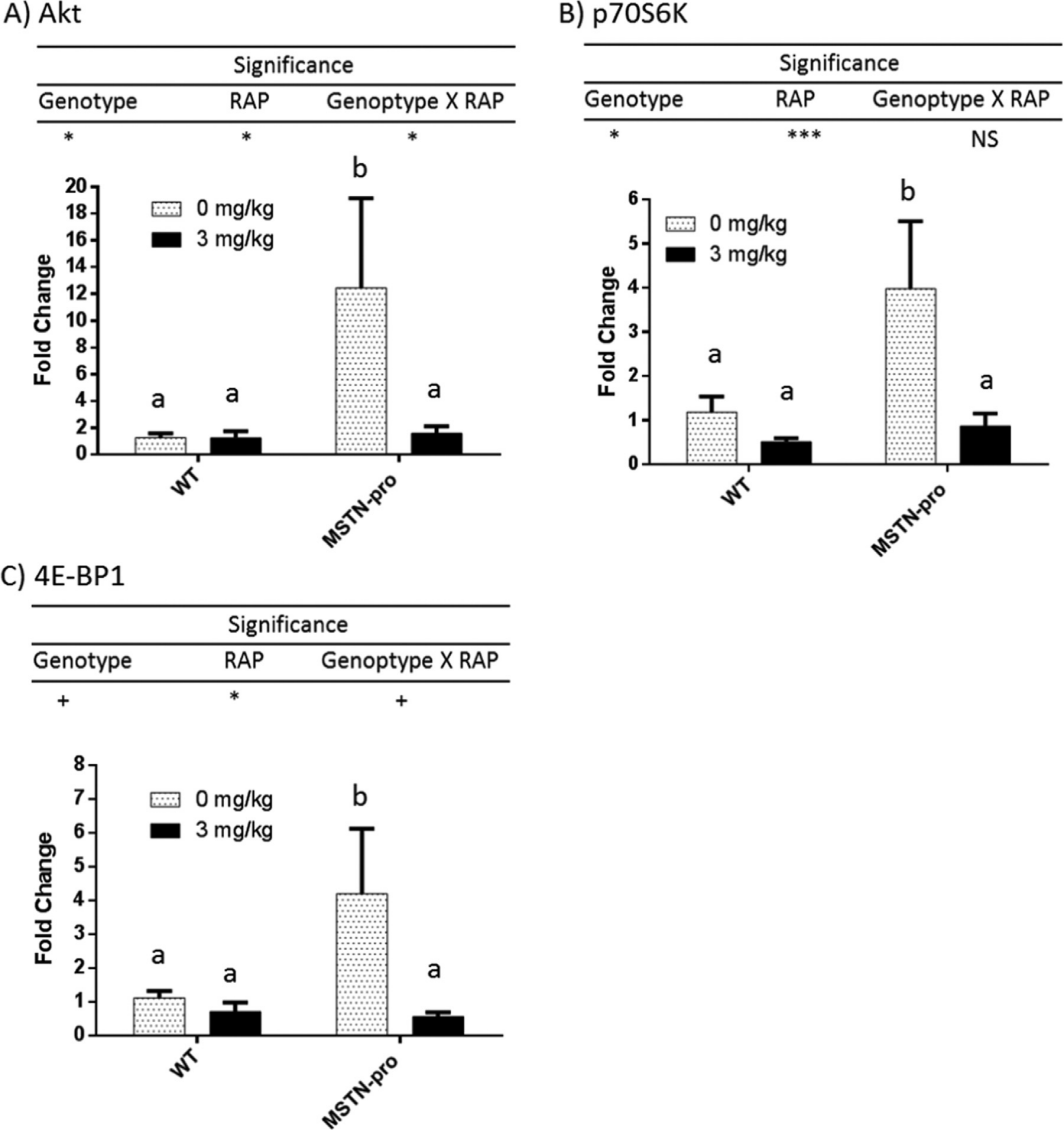
Rapamycin administration affects the expression of Akt, p70S6K1, 4E-BP1genes in wild and MSTN-pro type. Gene expression was measured from individual muscle samples. ΔCt values were calculated by subtracting respective CT value of GAPDH from the CT values of the target gene, then ΔΔCt values were calculated by subtracting mean ΔCt value of target gene from wild-type mice, followed by fold change calculation using the 2^-ΔΔCt^ method. The fold changes are expressed as mean ± (SEM). NS, not significant; **, P < 0.01; *, P < 0.05; NS, not significance. RAP, rapamycin. In addition to Two-way ANOVA, means of each group were also compared by the Tukey's multiple comparison test. The means not sharing the same superscript differ at P < 0.05.

The mRNA abundance of Akt, p70S6K, and 4E-BP1 in MSTN-pro and wild-type mice as affected by RAP is shown in Fig. 4. MSTN-pro mice had significantly higher mRNA abundances of Akt, p70S6K, and 4E-BP1 than those of wild-type mice. RAP administration suppressed the mRNA abundances Akt, p70S6K, and 4E-BP1 only in MSTN-pro mice, but not in wild-type mice.

The Akt/mTOR pathway plays a critical role in postnatal skeletal muscle hypertrophy by regulating muscle protein synthesis, and suppression of this pathway by RAP ether prevented muscle hypertrophy or induced muscle atrophy [49], [50]. The regulation of protein synthesis is primarily mediated by two well-known downstream effectors of the Akt/mTOR pathway, translational suppressor 4E-BP1 and ribosomal protein p70S6K [49], [50]. The principal mechanism for activating the Akt/mTOR pathway mostly involves the phosphorylation of its component molecules, and many studies reported that skeletal muscle hypertrophy induced by resistant exercise was associated with increased phosphorylations of signaling molecules of the Akt/mTOR pathway, including 4E-BP1 and p70S6K, without much effect on the total levels of these proteins [51], [52], [53], [54]. Bodine et al. [19], however, reported that total amount of Akt protein also increased (4 fold) together with phosphorylation of the protein (9 fold) during compensatory muscle hypertrophy of rat plantaris muscle, suggesting a presence of transcriptional regulation of the Akt/mTOR pathway as well. Many studies also examined the level of phosphorylation of component molecules of the Akt/mTOR pathway in association with MSTN-dependent muscle growth. Akt phosphorylation was suppressed by MSTN administration in human primary myotube and C2C12 derived myotubes [20]. Transient genetic overexpression of MSTN in rat muscles decrease the phosphorylation of various signaling molecules responsible for the Akt/mTOR pathway, including Akt, TSC2, p70S6K, and 4E-BP1 [21]. These studies together indicate that MSTN suppresses the Akt/mTOR signaling pathway by post-translational modulation (i.e., phosphorylation) of involved signaling molecules. Our current results indicate that inhibition of MSTN modulates the Akt/mTOR pathway at the transcriptional level in postnatal muscles as was shown by the significant increase (3–8 fold) in mRNA abundances of Akt, 4E-BP1 and p70S6K in MSTN-pro mice in comparison to wild-type mice. In support of our findings, genetic loss of MSTN in mice increased Akt gene expression as well as protein levels of Akt in postnatal muscles [19], [22]. These results together indicate that MSTN can modulate signaling molecules of the Akt/mTOR pathway at the transcriptional level as well as at the post-translational level in postnatal muscles.

Since RAP suppresses mTOR, activities of its downstream effectors are expected to be suppressed by RAP. Studies have shown that chronic administration of RAP suppresses the phosphorylation of p70S6K in rat skeletal muscles [30], [31], murine cardiac muscle [34], and murine liver [32]. Few studies, however, examined the effect of RAP on the mTOR signaling at the transcriptional level. Our current results showed that RAP administration suppressed the mRNA abundance of p70S6K and 4E-BP1 in MSTN-pro mice only, suggesting a down-regulation of these signaling molecules at the transcription level, particularly in conditions where muscle hypertrophy is induced by strong anabolic signals such as MSTN suppression. While little is known whether RAP is involved in transcriptional regulation of Akt, studies indicate that RAP plays a role in the regulation of Akt activity [30], [32], [55]. For example, chronic administration of RAP suppressed the phosphorylation of Akt in skeletal muscles of mice [32] and rat [30]. In C2C12 cell culture, RAP suppressed myotube hypertrophy and Akt phosphorylation [55]. Interestingly, our current results showed that RAP significantly suppressed mRNA abundance of Akt only in MSTN-pro mice. This result suggests that Akt transcription can be affected by RAP administration, and it remains to be determined what factors are involved in the differential response in Akt transcription by RAP between the MSTN-pro and wild-type mice. Presumably, the suppression of Akt expression induced a much far greater suppression of the Akt/mTOR pathway by RAP administration in MSTN-pro mice than in wild-type mice. Then it is speculated that the much greater suppression of muscle mass by RAP administration in MSTN-pro mice is related to the greater suppression of the Akt/mTOR pathway.

We did not measure the protein levels of Akt, p70S6K and 4E-BP1 or the phosphorylation of these proteins in the current study, so it remains to be examined whether the transcriptional down-regulation lowers protein levels of these molecules. Thus, the limitation of this study is that it is not clear whether the changes in mRNA abundance of Akt, p70S6k or 4E-BP1 resulted in changes in protein level and subsequent phosphorylation level of those molecules due to the lack of data on these measurements. Nevertheless, our current results show that genetic suppression of MSTN can enhance the Akt/mTOR pathway at the transcriptional level, and RAP can suppress the Akt/mTOR pathway also at the transcriptional level.

## 4 Conclusion

Rapamycin, a mTOR suppressor, suppressed skeletal muscle growth dramatically in MSTN-pro mice having a depressed MSTN function by its propeptide transgene as compared to wild-type mice. The suppression of muscle growth was accompanied by transcriptional suppression of Akt, p70S6k, and 4E-BP1, suggesting that MSTN suppresses skeletal muscle hypertrophy at least in part by inhibition of the Akt/mTOR pathway at the transcriptional level in addition to post-translational level. Additional new finding from this study showed that mRNA level of transcription factor Mrf4 in MSTN-pro mice was significantly lower than that of wild-type mice, suggesting that MSTN influences postnatal skeletal muscle growth via transcriptional regulation of Mrf4 gene.
