# Supplementary material for: Rapamycin suppresses postnatal muscle hypertrophy induced by myostatin-inhibition accompanied by transcriptional suppression of the Akt/mTOR pathway
Source: Biochem Biophys Rep. 2019 Jan 21;17:182–90. doi: 10.1016/j.bbrep.2018.12.009 (PMC6362869; doi:10.1016/j.bbrep.2018.12.009)
Supplement: Supplementary file 2 — Supplementary material [file mmc2.docx]

ranged between 22 – 32.

| **Gene** | **RefSeq** | **Primers (5’-3’)** | | **Tm** | **Amplicon**  **Size (bp)** |
| --- | --- | --- | --- | --- | --- |
| Gapdh | NM_001289726 | Forward | ACCCAGAAGACTGTGGATGG | 59.0 | 171 |
|  |  | Reverse | CACATTGGGGGTAGGAACAC | 58.2 |  |
| MyoD | NM_010866 | Forward | GACAGGGAGGAGGGGTAGAG | 60.1 | 219 |
|  |  | Reverse | TGCTGTCTCAAAGGAGCAGA | 59.0 |  |
| MyoG | NM_031189 | Forward | CTGCCTAAAGTGGAGATCCTG | 57.8 | 149 |
|  |  | Reverse | TGGGAGTTGCATTCACTGG | 57.7 |  |
| Mrf4 | NM_008657 | Forward | CCCTACAGCTACAAACCCAAG | 58.3 | 146 |
|  |  | Reverse | GCTGAGGCATCCACGTTTG | 59.5 |  |
| Myf5 | NM_008656 | Forward | AGGAAAAGAAGCCCTGAAGC | 58.1 | 151 |
|  |  | Reverse | GCAAAAAGAACAGGCAGAGG | 57.6 |  |
| Akt | NM_001331107 | Forward | GCCCTCAAGTACTCATTCCAG | 58.1 | 142 |
|  |  | Reverse | ACACAATCTCCGCACCATAG | 57.7 |  |
| P70S6k1 | NM_001114334 | Forward | TGAGTCAAGCCTTGGTCGAG | 59.7 | 125 |
|  |  | Reverse | AAGAGTCGAGAGAGACGCCC | 61.0 |  |
| 4E-BP1 | NM_007918 | Forward | CGGAAGATAAGCGGGCAG | 58.0 | 149 |
|  |  | Reverse | CAGTGTCTGCCTGGTATGAG | 57.7 |  |
| Myostatin | NM_010834 | Forward | TGCAAAATTGGCTCAAACAG | 55.6 | 182 |
|  |  | Reverse | GCAGTCAAGCCCAAAGTCTC | 59.1 |  |

**Table 1. Sequence of PCR primers used for real-time PCR**
